# Effects of IGF‐1 isoforms on muscle growth and sarcopenia

**DOI:** 10.1111/acel.12954

**Published:** 2019-04-05

**Authors:** Francesca Ascenzi, Laura Barberi, Gabriella Dobrowolny, Aline Villa Nova Bacurau, Carmine Nicoletti, Emanuele Rizzuto, Nadia Rosenthal, Bianca Maria Scicchitano, Antonio Musarò

**Affiliations:** ^1^ DAHFMO‐Unit of Histology and Medical Embryology Laboratory affiliated to Istituto Pasteur Italia—Fondazione Cenci Bolognetti Sapienza University of Rome Rome Italy; ^2^ School of Physical Education and Sport University of São Paulo São Paulo Brazil; ^3^ Department of Mechanical and Aerospace Engineering Sapienza University of Rome Rome Italy; ^4^ Imperial Centre for Translational and Experimental Medicine Imperial College London London UK; ^5^ The Jackson Laboratory Bar Harbor Maine; ^6^ Istituto di Istologia e Embriologia Università Cattolica del Sacro Cuore Fondazione Policlinico Universitario Agostino Gemelli Rome Italy

**Keywords:** aging, autophagy, IGF‐1, NMJ, sarcopenia, skeletal muscle

## Abstract

The decline in skeletal muscle mass and strength occurring in aging, referred as sarcopenia, is the result of many factors including an imbalance between protein synthesis and degradation, changes in metabolic/hormonal status, and in circulating levels of inflammatory mediators. Thus, factors that increase muscle mass and promote anabolic pathways might be of therapeutic benefit to counteract sarcopenia. Among these, the insulin‐like growth factor‐1 (IGF‐1) has been implicated in many anabolic pathways in skeletal muscle. IGF‐1 exists in different isoforms that might exert different role in skeletal muscle. Here we study the effects of two full propeptides IGF‐1Ea and IGF‐1Eb in skeletal muscle, with the aim to define whether and through which mechanisms their overexpression impacts muscle aging. We report that only IGF‐1Ea expression promotes a pronounced hypertrophic phenotype in young mice, which is maintained in aged mice. Nevertheless, examination of aged transgenic mice revealed that the local expression of either IGF‐1Ea or IGF‐1Eb transgenes was protective against age‐related loss of muscle mass and force. At molecular level, both isoforms activate the autophagy/lysosome system, normally altered during aging, and increase PGC1‐α expression, modulating mitochondrial function, ROS detoxification, and the basal inflammatory state occurring at old age. Moreover, morphological integrity of neuromuscular junctions was maintained and preserved in both MLC/IGF‐1Ea and MLC/IGF‐1Eb mice during aging. These data suggest that IGF‐1 is a promising therapeutic agent in staving off advancing muscle weakness.

## 1 INTRODUCTION

The progressive age‐related decline in skeletal muscle mass and strength, responsible for impaired mobility and disability in elderly, is referred as sarcopenia, and it is the result of multiple molecular and cellular changes. The decline in muscle mass involves a fiber number reduction of 30%–40% (Lexell, 1995), and a decrease in fiber size, with 10%–40% smaller type II fibers in elderly compared with young people (Frontera et al., 2000). Aging is also associated with a fast‐to‐slow fiber type shift due to the age‐dependent remodeling of motor units (Ciciliot, Rossi, Dyar, Blaauw, & Schiaffino, 2013; D'Antona et al., 2003; Delbono, 2011). At the cellular level, aging is caused by a progressive decline in mitochondrial function, resulting in accumulation of reactive oxygen species (ROS). The imbalance between protein synthesis and protein degradation and changes in metabolic and hormonal status, as well as in circulating levels of inflammatory mediators, have been also postulated as the key factors contributing to sarcopenia (Doherty, 2003; Fielding et al., 2011). Moreover, the accumulation of damaged macromolecules and cellular components, that constantly cause the initiation of an immune response, has been proposed as a potent contributor of the basal chronic inflammation associated with aging. Indeed, age‐related failure of removal of dysfunctional protein and organelles (Terman & Brunk, 2006), through autophagic pathways, is detrimental for muscle tissue and is likely correlated to sarcopenia (Sandri et al., 2013).

Among pathways that regulate protein turnover and muscle function, insulin‐like growth factor 1 (IGF‐1) plays a central role in muscle growth, differentiation, and regeneration (Scicchitano, Rizzuto, & Musarò, 2009). In the adult mammals, IGF‐1 is principally synthesized in the liver, acting as a systemic growth factor; however, it is also produced in extrahepatic tissues, including skeletal muscle, where it plays a mainly autocrine/paracrine role. The IGF‐1 protein is produced by different pre‐pro‐peptides, whereas two different promoters and differential splicing of the IGF‐1 gene create several IGF‐1 isoforms, which differ in the N‐terminal signal peptide (Class 1 or 2) and the C‐terminal extension peptide (E‐peptide Ea or Eb) (Shavlakadze, Winn, Rosenthal, & Grounds, 2005). Whether different IGF‐1 isoforms exert different biological functions, or whether existence of these isoforms reflects a mechanism for a tissue specific regulation of IGF‐1 expression is still unclear. IGF‐1Ea, the dominant IGF‐1 isoform, promotes satellites cell differentiation and provides most of the mature IGF‐1 for stimulating protein synthesis, whereas IGF‐1Eb is responsible for satellite cell activation and proliferation. It has been previously demonstrated that muscle overexpression of IGF‐1Ea isoform induced muscle hypertrophy in adulthood and guaranteed a maintenance of muscle mass and functionality during aging and in animal models of neuromuscular diseases (Barton‐Davis, Shoturma, Musaro, Rosenthal, & Sweeney, 1998; Bosch‐Marcé et al., 2011; Musarò et al., 2004,2001; Palazzolo et al., 2009). A recent study demonstrated that only full‐length IGF‐1Eb, but not Eb peptide alone, was able to promote anabolic effects on muscle (Fornaro et al., 2014); conversely, Eb peptides, without the influence of additional IGF‐1, were able to induce a significant muscle hypertrophy, which was surprisingly associated with a loss of muscle strength (Brisson, Spinazzola, Park, & Barton, 2014). Given the conflicting and still unclear data on effects of different IGF‐1 isoforms, we propose to study the function of the full‐length Class1_IGF‐1 Ea and Class1_IGF‐1Eb isoforms in skeletal muscle, with the purpose to investigate whether the overexpression of either propeptides IGF‐1Ea or IGF‐1Eb isoform impacts muscle aging and through which mechanisms each isoform acts. By expressing the different isoforms under the control of the same postmitotic skeletal muscle regulatory elements, we directly compared their effects in order to define whether they share or not a common pathway for their actions.

## 2 RESULTS

### 2.1 Effects of IGF‐1 isoforms overexpression on muscle growth and hypertrophy

**Figure 1:**
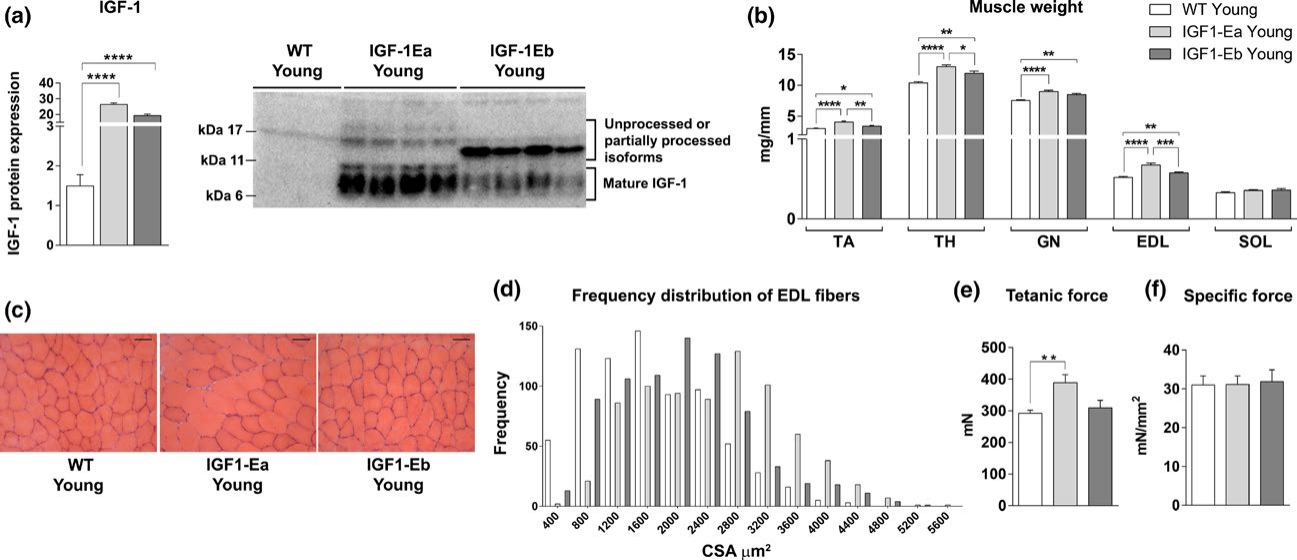
Effect of IGF‐1 isoforms on skeletal muscle weight in 6‐month‐old mice. (a) Densitometric analysis and representative Western blot bands of IGF‐1 protein; (b) Wet weight (mg), normalized for tibial length (mm), of fast (TA, TH, GN,, and EDL) and slow (SOL) skeletal muscles from wt and tg animals. (c) Hematoxylin and eosin‐stained cross sections of EDL from wt and tg mice (6 months old). Bar = 50 um. (d) Frequency distribution of cross‐sectional area (CSA) of extensor digitorum longus (EDL) muscles from the different experimental groups. Values of mean ± *SEM*: wt = 1673.42 ± 30.16; IGF‐1Ea = 2502.75 ± 35.29; IGF‐1Eb = 2016.35 ± 32.97. (e,f) Physiological properties of EDL muscles from tg and wt mice. All measurements are presented as mean ± *SEM*. *between young (6 months) wt and tg mice (*p < 0.05; **p < 0.001; ***p < 0.005; ****p < 0.0001 by Mann–Whitney *U* test)

We analyzed and compared the ability of muscle‐specific expression of full propeptides IGF‐1Ea and IGF‐1Eb (Supporting Information Figure S1a) to induce muscle hypertrophy and counteract sarcopenia. Real‐time PCR analyses demonstrated that each transgenic mouse line, namely IGF‐1Ea and IGF‐1Eb, up‐regulated selectively its specific class and peptide, without any compensatory modulation of other IGF‐1 isoforms (Supporting Information Figure S1b). Western blot analyses confirmed the up‐regulation of IGF‐1 protein in transgenic muscle compared to wild‐type and revealed two different IGF‐1 expression patterns in both transgenic mice (Figure 1a). The IGF‐1Ea muscle predominantly expressed the mature form of IGF‐1, whereas the IGF‐1Eb muscle displayed the unprocessed form. Of note, despite the different patterns, the total expression levels of IGF‐1, including mature partially processed and unprocessed isoforms, were comparable in the two models. As expected, only a faint band of IGF‐1 protein, corresponding to the IGF‐1Ea predominantly expressed isoform in muscle (Kern et al., 2011; Sandri et al., 2013), was detectable in wild‐type mice. Moreover, the muscle expression of either IGF‐1Ea or IGF‐1Eb transgene did not lead to systemic up‐regulation of the circulating IGF‐1 isoform (Supporting Information Figure S1c).

We then explored whether IGF‐1Eb, similarly to IGF‐1Ea (Musarò et al., 2001), is able to promote muscle hypertrophy. For this purpose, we analyzed, in wild‐type and IGF‐1Ea and IGF‐1Eb mice, the weight of different muscles such as quadriceps (TH), gastrocnemius (GN), tibialis anterior (TA), extensor digitorum longus (EDL), and Soleus (SOL). Of note, the fast‐fiber‐specific effects of the IGF‐1Ea and IGF‐1Eb transgenes were reflected in the increased average weight of muscles rich in fast fibers, compared with wild‐type muscles (Figure 1b). Histological analysis (Figure 1c) and frequency distribution of cross‐sectional area (CSA) (Figure 1d) of EDL myofibers revealed a shift of the median values toward large myofibers size in muscles of both transgenic mouse lines, compared to wild‐type littermates, although the hypertrophic muscle phenotype was more pronounced in IGF‐1Ea mice, compared to IGF‐1Eb animals.

To define whether the different levels of muscle hypertrophy, exerted by the two isoforms of IGF‐1, were associated with an increase in muscle performance, we analyzed the functional properties of IGF‐1Ea, IGF‐1Eb, and wild‐type muscles. Of note, only the increased muscle mass in IGF‐1Ea transgenic muscle was associated with increased force generation compared with both IGF‐1Eb and wild‐type muscles (Figure 1e,f).

### 2.2 Effect of IGF‐1 isoforms overexpression on skeletal muscle aging

**Figure 2:**
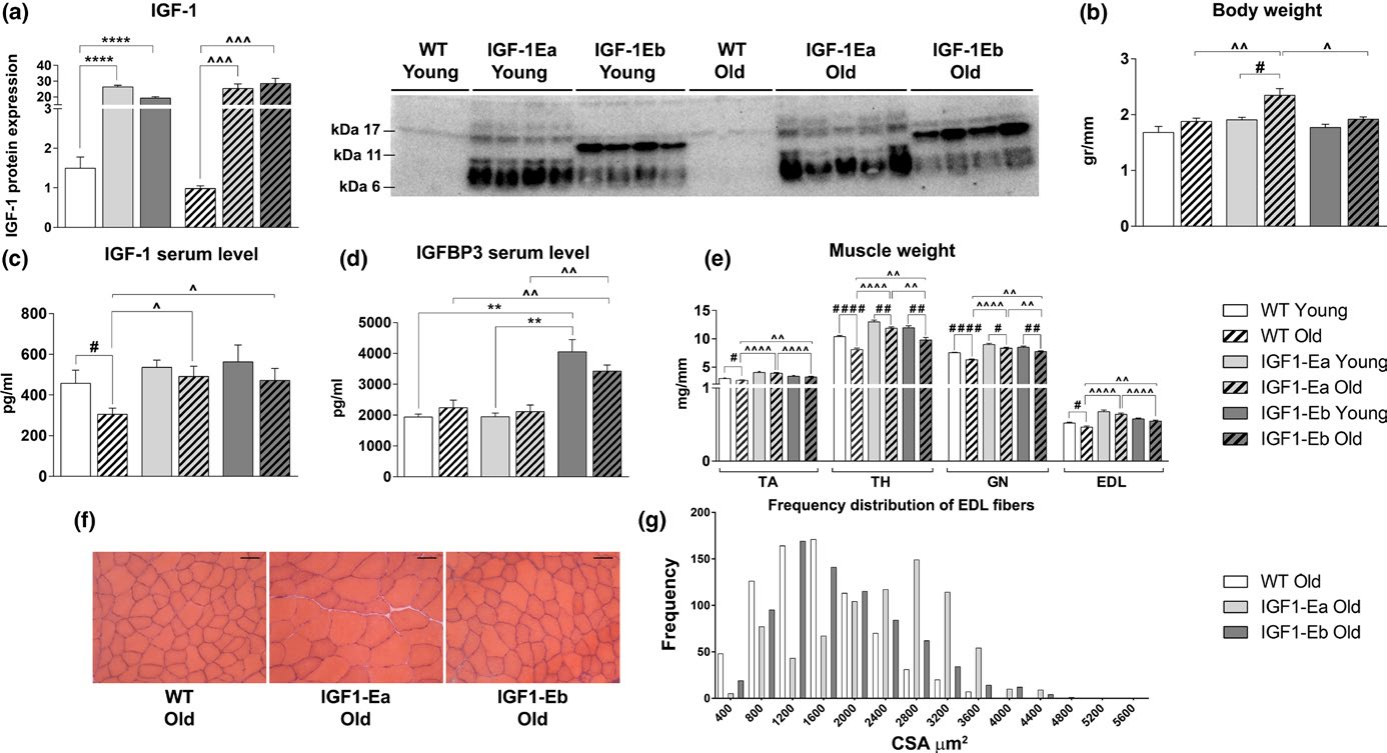
Analysis of the effects of IGF‐1 isoforms during aging. (a) Densitometric analysis and representative Western blot bands of IGF‐1 protein (the representative western blot related to young mice is part of the experiment also reported in Figure 1a); (b) body weight (gr), (c) serum levels of IGF‐1 (pg/ml), and (d) IGFBP‐3 (pg/ml); (e) skeletal muscle wet weight (mg), normalized for tibial length (mm), in wt and tg mice of young (6 months old) and aged (26 months old) mice. (f) Hematoxylin and eosin‐stained cross sections of EDL muscle from old wt and tg mice. (g) Frequency distribution of the myofibers cross‐sectional area (CSA) of extensor digitorum longus (EDL) from young and old wt and tg mice. Bar = 50 μm. Values of mean ± *SEM*: wt = 1554.00 ± 24.72; IGF‐1Ea = 2360.99 ± 31.80; IGF‐1Eb = 1790.32 ± 29.62. #between young and old mice in each group; ^between old wt and tg mice (#, ^*p* < 0.05; ##, ^^, **p < 0.005; ###, ^^^*p* < 0.001; ####, ^^^^, ****p < 0.0001 by Mann–Whitney *U* test). In (e), the statistical analysis between young wt and tg mice is not shown; it is indicated in Figure 1b

In order to study the effects of IGF‐1 isoforms overexpression during aging, we analyzed IGF‐1Ea and IGF‐1Eb transgenic mice at 26 months of age, comparing them with age‐matched wild‐type mice. Western blot analyses demonstrated that the up‐regulation of IGF‐1 protein was maintained during aging without changes in the expression pattern (Figure 2a). We observed that only aged IGF‐1Ea mice displayed significant increase in body weight compared to both age‐matched wild‐type and IGF‐1Eb mice (Figure 2b). To exclude systemic effects of long‐term overexpression of IGF‐1 transgenes, we analyzed serum levels of IGF‐1 and the weight of total body and visceral organs of young and aged mice. The evaluation of visceral organs weight and the morphologic and molecular analyses of the cardiac tissue revealed that skeletal muscle‐specific expression of IGF‐1 isoforms caused no significant changes in transgenic mice compared to wild type littermates (data not shown). Interestingly, consistent with the physiological decline in serum IGF‐1 during aging (Landin‐Wilhelmsen, 2004), we observed a strong reduction of serum IGF‐1 levels in old wild‐type mice, whereas aged transgenic animals exhibited unchanged levels of IGF‐1 compared to young counterparts (Figure 2c).

We then analyzed the expression levels of relevant circulating IGFBPs, namely IGFBP‐2 and IGFBP‐3 in young and aged wild‐type and IGF‐1Ea and IGF‐1Eb transgenic mice. While IGFBP‐2 levels did not significantly change between wild‐type and transgenic animals (data not shown), we observed a significant increase in circulating protein levels of IGFBP‐3 in both 6‐month‐old and 26‐month‐old IGF‐1Eb mice, compared to wild‐type and IGF‐1Ea age‐matched animals (Figure 2d). Of note, circulating IGFBP‐3 levels did not display any significant change in IGF‐1Ea transgenic mice compared to wild‐type littermates.

We then verified whether expression of the IGF‐1Eb isoform, similarly to IGF‐1Ea and despite the lack of functional muscle hypertrophy, was able to counteract the decline in muscle mass during aging. We observed an increase in weight of different muscles from transgenic mouse models compared to wild‐type littermates (Figure 2e). As shown by the representative H&E images (Figure 2f) and by the CSA frequency distribution graph (Figure 2g), myofibers from EDL of aged IGF‐1Ea and IGF‐1Eb transgenic mice were significantly bigger than those of wild‐type mice. Notably, muscles of IGF‐1Ea mice were characterized by increased weight and CSA values compared with those of IGF‐1Eb littermates.

**Figure 3:**
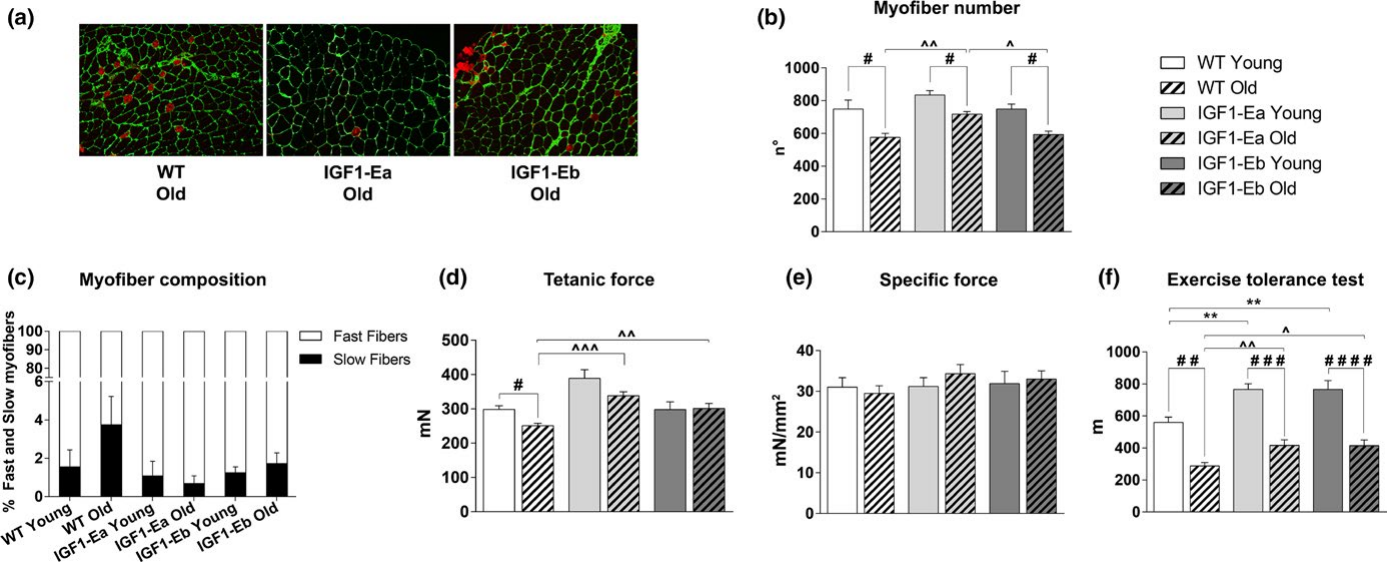
Analysis of the effect of IGF‐1 isoforms on fiber type composition and muscle strength in aging. (a) Immunofluorescence of transverse sections from EDL of aged (26 months old) wild‐type and IGF‐1Ea and IGF‐1Eb transgenic mice (*n* = 3 per group), stained with antibodies against slow myosin (red) and laminin (green). (b) Myofibers number of EDL muscle from young and old wt and tg mice. (c) The mean number of fast (white) and slow (black) myofibers is expressed as percentage of total number of myofibers in each experimental group (wt young 1.56 ± 0.88; wt old 3.75 ± 1.47; IGF‐1Ea young 1.08 ± 0.76; IGF‐1Ea old 0.68 ± 0.40; IGF‐1Eb young 1.25 ± 0.30; IGF‐1Eb old 1.72 ± 0.56). (d,e) Physiological properties of EDL muscles from young and aged wild‐type and transgenic mice. (f) Maximum running distance (m) determined by exhaustion of all analyzed mice on the ETT. All measurements are presented as mean ± *SEM*. #between young and old mice in each group; ^between old wt and tg mice (#, ^*p* < 0.05; ##, ^^, **p < 0.005; ###, ^^^*p* < 0.001; ####, ^^^^*p* < 0.0001 by Mann–Whitney *U* test). In (d,e), the statistical analysis between young (6 months) wt and tg mice is not shown; it is indicated in Figure 1e,f

To strengthen evidence that muscle expression of IGF‐1 isoforms is able to counteract muscle aging, we performed additional analyses evaluating the degree of muscle wasting. Analysis of EDL myofiber numbers revealed that IGF‐1Ea isoform more efficiently protects skeletal muscle from the fiber loss occurring in aging, compared to both aged wild‐type and IGF‐1Eb mice (Figure 3b). Indeed, 26‐month‐old IGF1Ea mice displayed a reduction in muscle fibers number of about 14% (13.99% ± 1.98) compared to young IGF‐1Ea mice, whereas both aged wild‐type (23% ± 3.28) and IGF‐1Eb mice (20.84% ± 2.86) showed a significant reduction in muscle fibers compared to the respective young counterparts. We then analyzed the percentage of both fast and slow fibers in wild‐type and transgenic mice at the different age (Figure 3a,c). While aged wild‐type mice displayed an increased, not statistically significant, percentage of slow myofibers with a reduction in fast ones, the expression of either IGF‐1Ea or IGF‐1Eb isoform maintained the fiber type composition during aging. Moreover, independently of the extent of increased muscle mass, both aged IGF‐1Ea and IGF‐1Eb mice showed an increase in force generation, compared with age‐matched wild‐type littermates (Figure 3d,e).

In order to evaluate the whole physical performance, wild‐type and transgenic mice of different ages were subjected to the exercise tolerance test (ETT), revealing that both young and aged IGF‐1Ea and IGF‐1Eb transgenic mice were able to significantly increase running distance compared with age‐matched wild‐type (Figure 3f). It was also interesting to note that IGF‐1Eb mice, despite a lower muscle mass compared to IGF1‐Ea mice, showed a similar physical performance.

### 2.3 Analysis of molecular markers of protein synthesis and degradation

To determine the mechanisms activated by IGF‐1 isoforms to mitigate aging‐dependent muscle wasting, we analyzed relevant markers of anabolic and catabolic pathways, such as Akt kinase and its downstream effector rapamycin (mTOR) (Schiaffino, Dyar, Ciciliot, Blaauw, & Sandri, 2013), along with the muscle‐specific atrophy‐related ubiquitin ligases gene, namely Atrogin‐1 and MuRF1, as well as markers of the autophagic pathways (Sandri et al., 2013).

**Figure 4:**
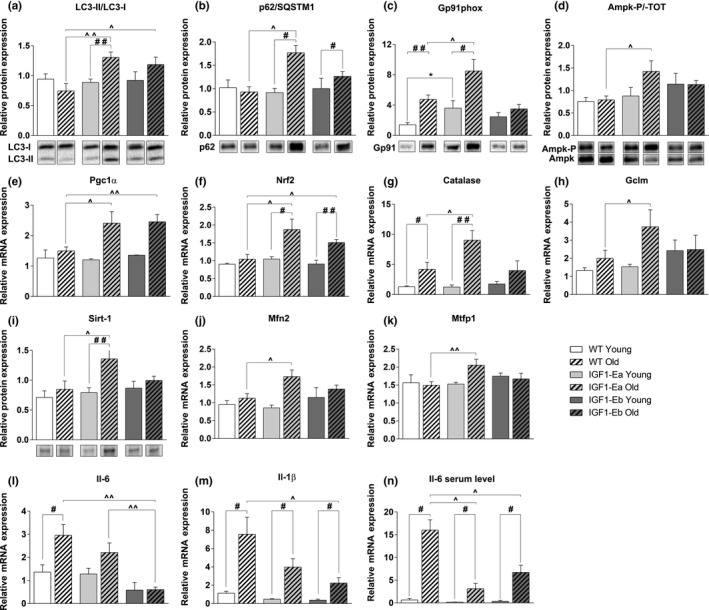
Effect of IGF‐1 isoforms on different molecular pathways during aging. Densitometric analyses (upper panel) and representative Western blot bands (lower panel) in TA muscle from young (6 months old) and aged (26 months old) wt and tg mice for the expression of: LC3 (a) (analysis of the ratio of LC3‐II/LC3‐I, normalized to the total protein); SQSTM1/p62 (b), gp91 (phox) (c), phospho‐AMPK (Thr172) and total AMPK (d) (analysis of the ratio between the phosphorylated and the total protein), and SIRT‐1 (i). The representative bands come from not contiguous lanes in the same gel. Values are reported as protein content relative to that of young wt mice. Real‐time PCR analysis for the expression of PGC‐1α (e), NRF‐2 (f), Catalase (g), GCLM (h), MFN2 (j), MTFP1 (k), IL‐6 (l), and IL‐1β (m) in TH muscles from young (6 months old) and aged (26 months old) wt and tg mice. (n) Serum levels of IL‐6 (pg/ml). Values are reported as fold change in expression and represent mean ± *SEM*; *n* = 4–8 per group. *between young wt and tg mice; #between young and old mice in each group; ^between old wt and tg mice (*, #, ^*p* < 0.05; **, ##, ^^*p* < 0.005; ***, ###, ^^^*p* < 0.001; ****, ####, ^^^^*p* < 0.0001 by Mann–Whitney *U* test)

Western blot analysis did not reveal any significant modulations of Akt and mTOR phosphorylation between the different mouse models and during aging (data not shown). Instead, RNA expression levels of the atrogenes markedly increased in all aged animal models except for atrogin‐1 that did not change significantly between young and aged transgenic mice (Supporting Information Figure S1d,e). Western blot analyses of the relevant markers of the autophagic pathway, normally altered during aging (Carnio et al., 2014), revealed a significant up‐regulation of the LC3‐II/LC3‐I protein ratio (Figure 4a) and of the autophagosome cargo protein p62/SQSTM1(Figure 4b) in the muscles of both aged IGF‐1Ea and IGF‐1Eb mice compared to those of wild‐type littermates.

### 2.4 Analysis of oxidative stress and mitochondrial biogenesis

Another potential mechanism involved in the pathogenesis of sarcopenia is an enhancement of ROS production and the increase in oxidant damage (Carnio et al., 2014). We analyzed gP91 protein, a catalytic subunit of NOX2 that belongs to enzyme's family dedicated to ROS production. We observed a significant up‐regulation of this protein in the muscle of both young and aged IGF‐1Ea mice, but not in the muscle of either wild‐type or IGF‐1Eb mice (Figure 4c).

Of note, the increase in ROS production in the muscle of aged IGF‐1Ea mice was compensated with the modulation of relevant energy sensors, such as AMPK (Figure 4d), and of markers with antioxidant activity, namely PGC1‐α and Nrf‐2 (Figure 4e,f). Consistent with these data, we also found that the levels of mRNAs that encode the rate‐limiting enzyme for glutathione biosynthesis (glutamyl cysteine ligase modulator, GCLM) and catalase, a ROS scavenging enzyme, were significantly up‐regulated in old IGF‐1Ea, compared with wild‐type and IGF‐1Eb littermates (Figure 4g,h). Interestingly, the expression of Sirt1 protein, a factor that has broad biological functions in growth regulation, stress response, endocrine signaling, and extended lifespan (Kim & Um, 2008), resulted significantly up‐regulated in aged IGF‐1Ea mice, while its expression did not change between IGF‐1Eb and wild‐type muscle in both young and aged mice (Figure 4i).

In addition to the role in antioxidant defense, PGC1‐α has been implicated in mitochondrial dynamics by promoting the fusion and fission of mitochondria and ultimately regulating their quality and functionality (Dabrowska, 2015). We observed, similarly to PGC1‐α expression profile, increased mRNA levels of mitochondrial fusion protein MFN2, which is a PGC1‐α molecular target, in both aged IGF‐1Ea and IGF‐1Eb transgenic mice with respect to wild‐type aged‐matched mice (Figure 4j). Moreover, mRNA expression levels of MTFP1, a protein involved in mitochondrial fission, significantly increased in aged IGF‐1Ea mice and showed a moderate, not significant, up‐regulation in aged IGF‐1Eb mice compared to wild‐type littermates (Figure 4k). The significantly elevated expression of these markers in IGF‐1Ea mice suggests that this isoform preferentially promotes enhanced control of mitochondrial quality.

### 2.5 Analysis of inflammatory markers

Since there is growing recognition of the central role of increased low‐grade inflammatory response in aging‐sarcopenia and it has been reported that PGC1‐α downregulates inflammation (Dinulovic et al., 2016), we analyzed inflammatory cytokine expression in skeletal muscle from the different mice. Skeletal muscle from old wild‐type mice significantly up‐regulated IL‐6 and IL‐1b cytokine RNA expression, whose levels were strongly reduced in the muscle of both IGF‐1Ea and IGF‐1Eb aged mice (Figure 4l,m). Coherently, the analysis of circulating IL‐6 levels showed an increase in serum of aged mice and a significant reduction in both IGF‐1Ea and IGF‐1Eb transgenic mice compared to wild‐type littermates (Figure 4n).

### 2.6 IGF‐1 isoforms expression preserves NMJ integrity

**Figure 5:**
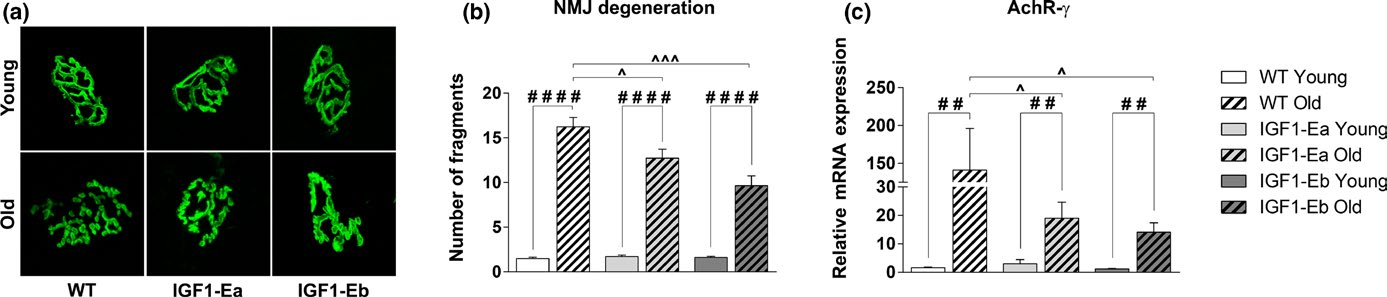
Analysis of the effect of IGF‐1 expression on the morpho‐functional changes of NMJs during aging. (a) Representative maximum projection of photomicrographs showing α‐bungarotoxin staining in longitudinal cryosections of TH muscle from aged (26 months old) wt and tg mice. (b) Mean number of fragments and/or discontinuity regions of α‐bungarotoxin staining in young and old, wt and tg mice. All measurements are presented as mean ± *SEM*; *n* = 3–5 mice per group. (c) Real‐time PCR analysis for the expression of gamma subunit of the Acetylcholine receptor. Values are reported as fold change in expression and represent mean ± *SEM*; *n* = 3–8 mice per group. *between young wt and tg mice; #between young and old mice in each group; ^between old wt and tg mice (*,#, ^*p* < 0.05; **, ##, ^^*p* < 0.005; ***, ###, ^^^*p* < 0.001; ****, ####, ^^^^*p* < 0.0001 by Mann–Whitney *U* test)

Since one of the mechanisms attributed to the loss of muscle mass during aging is a preceding myofiber denervation (Jang & Van Remmen, 2011), we aimed to determine whether the expression of different IGF‐1 isoforms would counteract age‐related neuromuscular junctions (NMJ) degeneration. Histological analysis, by α‐bungarotoxin staining on the longitudinal sections of quadriceps muscle, revealed marked alterations in the NMJ of aged wild‐type mice and not in age‐matched IGF‐1Ea and IGF‐1Eb mice (Figure 5a,b), suggesting that the expression of IGF‐1 isoforms counteracts NMJ fragmentation. To support this observation, we performed gene expression analysis for the gamma subunit of AChR (AChRγ), whose expression increases in denervated muscle or under conditions that alter the NMJ functionality (Witzemann, Brenner, & Sakmann, 1991). Real‐time PCR analysis revealed that AChRγ expression was dramatically up‐regulated during aging in the muscle of wild‐type mice, compared with that observed in both IGF‐1Ea and IGF‐1Eb littermates (Figure 5c).

## 3 DISCUSSION

In this study, we analyzed the effects of overexpression of full‐length Class 1 IGF‐1Ea and Class 1 IGF‐1Eb isoforms on muscle growth and their ability to counteract sarcopenia. Two major IGF‐1 transcripts are known: the locally acting isoform with an autocrine/paracrine action and the circulating isoform with endocrine effects (Scicchitano et al., 2009). The physiological differences between the function of local and circulating isoform of IGF‐1 are not completely established, and controversies exist between the activity of local/tissue specific production of IGF‐1 versus liver/circulatory IGF‐1. Injury of mammalian tissues induces transient production of locally acting IGF‐1 isoforms that control growth, survival, and differentiation. Contrarily, circulating IGF‐1 isoforms can induce hyperplasia and spontaneous tumor formation (Musaro & Rosenthal, 2006). Thus, the restricted action of supplemental IGF‐1 to muscle should guarantee the autocrine/paracrine role, excluding possible endocrine effects on other tissues. Moreover, it has been demonstrated that the insulin and IGF‐1 signaling pathway plays a critical role in the control of longevity in invertebrates; in contrast, their potential involvement in human longevity remains dubious. The reasons for the controversial action of insulin/IGF‐1 signaling between invertebrate and mammals can be numerous: among these, the different affinity of insulin, IGF‐1 and IGF‐2, to receptors and the activation of different metabolic pathways.

Our data demonstrated that muscle localized expression of either IGF‐1Ea or IGF‐1Eb counteracts sarcopenia, without inducing any evident sign of side effect in other tissues and organ and without limiting the lifespan of the animals. Preliminary results indicate that the two transgenic mouse models live longer compared to wild‐type mice.

It is plausible that local expression of IGF‐1 isoforms, preserving skeletal muscle, might maintain the youth not only of muscle tissue but also of the entire organism, as demonstrated by the exercise tolerance test (ETT). Interestingly, skeletal muscle has recently been identified as an endocrine organ capable to produce, express, and release, following exercise training, cytokines, and other peptides, known as myokines, that exert paracrine, autocrine, or endocrine effects. We can speculate that IGF‐1 is a sort of myokine that promotes a local effort for a global impact (Musarò, 2012), inducing health benefits. Thus, the reduction in circulating IGF‐1 levels observed in aged wild‐type mice could be the result of muscle morpho‐functional alterations; conversely, transgenic mice, guaranteeing the muscle expression levels of IGF‐1 isoforms even at late postnatal life, preserve the capability of muscle to function as endocrine organ, thus contributing to maintain unaltered the circulating IGF‐1 levels.

In a recent work, it has been addressed the question on whether the selective expression of Ea and Eb peptides on skeletal muscle is able to induce any specific anabolic effects even in the absence of mature IGF‐1 peptide (Brisson et al., 2014). The authors revealed that E‐peptides increase skeletal muscle mass but at the expense of strength, suggesting that a cooperative effect between E‐peptide and IGF‐1 mature peptide is necessary to promote more significant functional effects on skeletal muscle. Nevertheless, it remained to be determined whether full IGF‐1Ea and IGF‐1Eb propeptides exerted different role on muscle growth and homeostasis.

In previous works, we demonstrated that localized IGF‐1Ea transgene expression sustains hypertrophy and regeneration in senescent skeletal muscle (Musarò et al., 2001). However, to date it is not clear whether IGF‐1Eb isoform could outperform IGF‐1Ea isoform and whether the two isoforms activate different molecular mechanisms. Comparing the effects of the overexpression of two IGF‐1 isoforms on the skeletal muscle, we revealed that only IGF‐1Ea is able to promote a pronounced hypertrophic phenotype in adult mice, which is maintained in aged mice. In particular, muscles of IGF‐1Ea mice were characterized by increased muscle weight and CSA values than those of IGF‐1Eb. Although different molecular mechanisms can be involved in the induction and maintenance of the hypertrophic phenotype, it was interesting to note different expression pattern of IGF‐1 protein in IGF‐1Ea and IGF‐1Eb transgenic mice. We can hypothesize that the higher expression levels of mature IGF‐1 protein in IGF‐1Ea mice, compared to IGF‐1Eb one, may be responsible for the hypertrophic phenotype exerted by IGF‐1Ea isoform.

Nevertheless, beside the promotion of muscle growth, both IGF‐1Ea and IGF‐1Eb are able to counteract sarcopenia, activating pathways normally affected during aging, namely autophagy and PGC‐1‐mediated signaling (Sandri et al., 2013). These pathways control two important destabilizing factors associated with sarcopenia: the removal of dysfunctional mitochondria, responsible for excessive production of ROS, and the maintenance of NMJ integrity, essential to muscle function, and muscle‐nerve interplay.

Interestingly, we observed a significant up‐regulation of relevant molecular markers of the autophagic pathway in aged muscles of both IGF‐1Ea and IGF‐1Eb mice, compared to wild‐type mice. Our data also revealed that the activation of autophagic pathway underlies the ability of both isoforms of IGF‐1 to preserve the integrity and the morphology of NMJ during aging, protecting muscle fibers by denervation. In addition, the modulation of PGC‐1α, by IGF‐1Ea and IGF‐1Eb isoforms, emerges as a key aspect of the ability of the two IGF‐1 isoforms to counter sarcopenia. There is growing recognition of the central roles of inflammatory cytokines, such as IL‐6, in aging‐induced sarcopenic phenotypes (Visser et al., 2002). Here we reported the negative modulation of IL‐1β and IL‐6 levels by both IGF‐1Ea and IGF‐1Eb overexpression, counteracting the inflame‐aging process.

**Figure 6:**
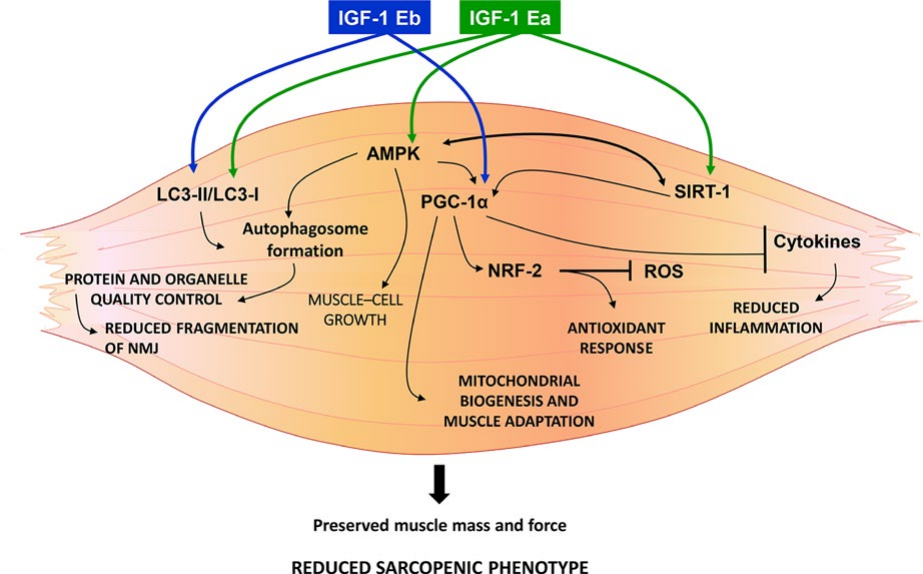
A summary of the molecular pathways responsible for the protective role of IGF‐1 isoforms against sarcopenia. Muscle localized expression of either IGF‐1Ea or IGF‐1Eb counteracts sarcopenia, through the coordinated activation of two pathways, namely PGC1α and autophagy. IGF‐1Ea or IGF‐1Eb activates the autophagy/lysosome system, normally altered during aging, to maintain the cell clear from dysfunctional organelles and mis‐ or unfolded proteins that are prone to aggregate during aging. PGC‐1α induces mitochondriogenesis and stimulates specific gene programs to guarantee the maintenance of muscle mass and muscle adaptation (i.e., fiber type specification, NMJ stability, and reduced inflammation). Notably, IGF‐1Ea expression stimulates additional pathways (i.e., AMPK, SIRT1) to guarantee a functional hypertrophic phenotype even during aging

The maintenance of hypertrophic phenotype by IGF‐1Ea involves also the activation of additional pathways, such as AMPK, a factor involved in the maintenance of whole‐body energy balance and an “energy sensor” controlling glucose and lipid metabolism (Kjøbsted et al., 2018). Indeed, it was up‐regulated in the muscle of IGF‐1Ea but not in IGF‐1Eb mice. The high metabolic rate observed in IGF‐1Ea muscle was correlated with high ROS production. These mice, however, were able to minimize oxidative damage in senescent muscle up‐regulating, through PGC1‐α activation, NRF‐2 protein, the master regulator of antioxidant defense (Scicchitano, Pelosi, Sica, & Musarò, 2018) and Sirt‐1, a factor involved in growth regulation, stress response, endocrine signaling, and extended lifespan. In aged IGF1‐Ea mice, the increased levels of markers involved in the fusion and fission of mitochondria could further contribute to the control of mitochondrial quality and functionality. Our data are consistent with a model (Figure 6) in which muscle expression of either IGF‐1Ea or IGF‐1Eb, activating a series of anabolic and compensatory pathways, is able to prevent muscle loss and a normal muscle‐nerve interaction, counteracting sarcopenia.

## 4 EXPERIMENTAL PROCEDURES

### 4.1 Generation of transgenic mice and aging colony

IGF‐1‐Eb isoform expression construct was generated by cloning the rodent cDNA sequence of Class 1_IGF‐1Eb into the skeletal muscle‐specific expression cassette (Musarò & Rosenthal, 1999) containing the myosin light chain MLC 1 promoter, a SV40 poly A signal and the MLC 1/3 enhancer sequence. FVB male mice (Jackson Laboratories) were used as embryo donors, and the transgenic animals were generated using standard methods. Positive founders for each IGF‐1Ea and IGF‐1Eb transgenic lines were subsequently bred to FVB wild‐type mice, and MLC/IGF‐1Ea and MLC/IGF‐1Eb transgenic mice were selected by PCR using tail digests. The animals were housed in individually ventilated cages (4‐5 per cage) in a temperature (22°C) and humidity (45%–55%) controlled room with a 12:12 hr light/dark cycle. All cages contained wood shavings, bedding, and a cardboard tube for environmental enrichment and spontaneous physical activities. Mice were provided with food (Teklad Global 18% Protein Rodent Diet (Envigo, Huntingdon, UK)) and water ad libitum. Sentinel mice were tested every four months to detect potential infection, analyzing the presence of viral, bacterial, and parasitic pathogens listed in the FELASA recommendations. The animals used for the experiments were specific pathogen free. Young (6 months of age) and old (26 months of age) wild‐type, IGF‐1Ea, and IGF‐1Eb heterozygous transgenic mice were used for all the experiments. In our study, we used male mice, based on the consideration that females, because their hormonal changes during aging, are subjected to a great individual variability, leading to confounder effects. Preliminary results on aged female revealed similar morpho‐functional and molecular signatures observed in male. Since we did not observe any significative differences in phenotypic features and in protein and gene expression levels among wild‐type mice and transgenic negative littermates for both IGF‐1Ea and IGF‐1Eb mice (Supporting Information Figure S2), we indicated as wild‐type the control mice. All experiments were conducted within the animal welfare regulations and guidelines.

### 4.2 Histological and immunofluorescence analysis

EDL muscles from wild‐type (wt) and transgenic (tg) mice were embedded in tissue freezing medium and snap‐frozen in nitrogen‐cooled isopentane. For histological and morphometric analysis, frozen cross sections (7 µm) were stained for hematoxylin and eosin. The distribution and the mean values of CSA, of at least 700 myofibers for each mice group, were analyzed with ImageJ software (Schneider, Rasband, & Eliceiri, 2012). For immunofluorescence analysis, EDL muscle sections were fixed with 4% PFA and processed as described (Dobrowolny et al., 2005). We used the following antibodies: anti‐Laminin, anti‐Myosin Slow, and anti‐Myosin Fast Abs (Sigma‐Aldrich, Saint Louis, MO, USA). Inverted microscope (Axioskop 2 plus; Carl Zeiss Micro Imaging, Inc., Jena Germany) was used, and images were processed using Axiovision 3.1. The number of fast and slow fibers was calculated as percentage of the total number of fibers in the cross section of EDL muscle of the different group of mice. For NMJ analysis, longitudinal frozen sections (40 μm) of quadriceps muscles were stained for fluorescent (BGT‐AF488) α‐bungarotoxin (Thermo Fisher Scientific, Waltham, MA, USA) and confocal images were analyzed using Leica Laser Scanning TCS SP2.

### 4.3 Protein extraction and Western blot analysis

Protein extraction was performed from TA muscles of wild‐type and transgenic mice at the different age. Muscles were homogenized in lysis buffer (Giusto et al., 2017), and equal amounts of protein from each muscle lysate were separated in SDS polyacrylamide gel (Criterion™ TGX Stain‐Free™ precast gel, Bio‐Rad, Hercules, CA, USA). For immunoblot were used antibodies against Phospho‐Akt (Thr308) (Sigma, Saint Louis, MO, USA); Akt, Phospho‐mTOR (Ser2448), mTOR, LC3B, Phospho‐AMPK (Thr172), AMPK (Cell Signaling Technology, Danvers, MA, USA); SQSTM1/p62, NOX2/gp91 phox (Abcam, Cambridge, GB); IGF‐1 (R&D system, Minneapolis, MN, USA); and Sirtuin1/SIRT1 (Novus Biologicals, Littleton, CO, USA). Signals were acquired by ChemiDoc™ MP Instrument (Bio‐Rad, Hercules, CA, USA), and densitometric analysis was performed using Image Lab acquisition analysis software (Image Lab Software Version 5.2.1). Each Western blot band intensity was normalized to stain‐free total lane protein. The expression levels of the analyzed proteins were calculated with respect to young control group and reported as mean fold change values.

### 4.4 RNA extraction and quantitative reverse transcription polymerase chain reaction (qPCR)

Total RNA from quadriceps (TH) muscles was extracted in Tri‐Reagent™ (Sigma, Saint Louis, MO, USA) using Tissue Lyser (Qiagen, Hilden, Germany). The reverse transcription reactions were performed with QuantiTect Reverse Transcription kit (Qiagen, Hilden, Germany), according to the manufacturer’s instruction. Gene expression levels were measured by quantitative real‐time PCR (qRT–PCR) in an ABI PRISM 7500 Sequence Detection System (Life Technologies, Carlsbad, CA, USA), using premade 6‐carboxyfluorescein (FAM)‐labeled TaqMan assays for Hprt1, Nfr2, PGC‐1α, IL‐6, IL‐1β, atrogin, MuRF1, catalase, Gclm, AchRγ, MFN2, MTFP1, and for the different IGF‐1 isoform sequences (Class I, Class II, Ea peptide, Eb peptide). Quantitative RT–PCR sample values were normalized to the expression of Hprt1 mRNA. The relative level for each gene was calculated using the 2‐ΔΔCT method (Livak & Schmittgen, 2001) and reported as mean fold change in gene expression.

### 4.5 ELISA analysis

To determine circulating IGF‐1 and IL‐6 levels, ELISA analysis was performed using mouse DuoSet ELISA kits (R&D system, Minneapolis, MN, USA). Serum levels of IGFBP‐2 and IGFBP‐3 were measured by mouse SimpleStep ELISA^®^ Kits (Abcam, Cambridge, UK).

### 4.6 Functional analysis

To test in vitro the muscle functional properties of wt and tg mice, the EDL muscle was isolated from the mice and transferred to a temperature‐controlled chamber (30°C) containing a Krebs‐Ringer solution equilibrated with 5% CO_2_–95% O_2_. Muscle contractions were electrically evoked by means of two platinum electrodes with 300 mA controlled current pulses, as detailed in Del Prete, Musarò, and Rizzuto (2008).

### 4.7 Exercise tolerance test (ETT)

After a period of acclimation to the treadmill, all mice at the different analyzed age began to run at initial speed of 6 m/min; speed was increased every 3 m/min. Maximum exercise tolerance and exhaustion were determined when the mouse became unable to run.

### 4.8 Statistics

Statistical analysis was performed with GraphPad Prism v6.0 software. All data are expressed as mean ± *SEM*. Groups were compared by nonparametric test (Mann–Whitney *U* test). A value of <0.05 was considered statistically significant.

## CONFLICT OF INTEREST

None declared.

## AUTHORS' CONTRIBUTION

AM and NR were responsible for conception and design of the study; FA, LB, GD, CN, ER, AB, and BMS contributed to data acquisition and statistical analysis; AM and NR contributed to the drafting of the text; CN, AB, and ER were responsible for functional analysis; FA and LB were responsible for data validation and figures preparation; FA, LB, GD, CN, ER, AB, BMS, and NR contributed to critical revision of the manuscript; AM finalized the text.
