# Supplementary figures and images for: Effects of IGF‐1 isoforms on muscle growth and sarcopenia
Source: Aging Cell. 2019 Apr 5;18(3):e12954. doi: 10.1111/acel.12954 (PMC6516183; doi:10.1111/acel.12954)

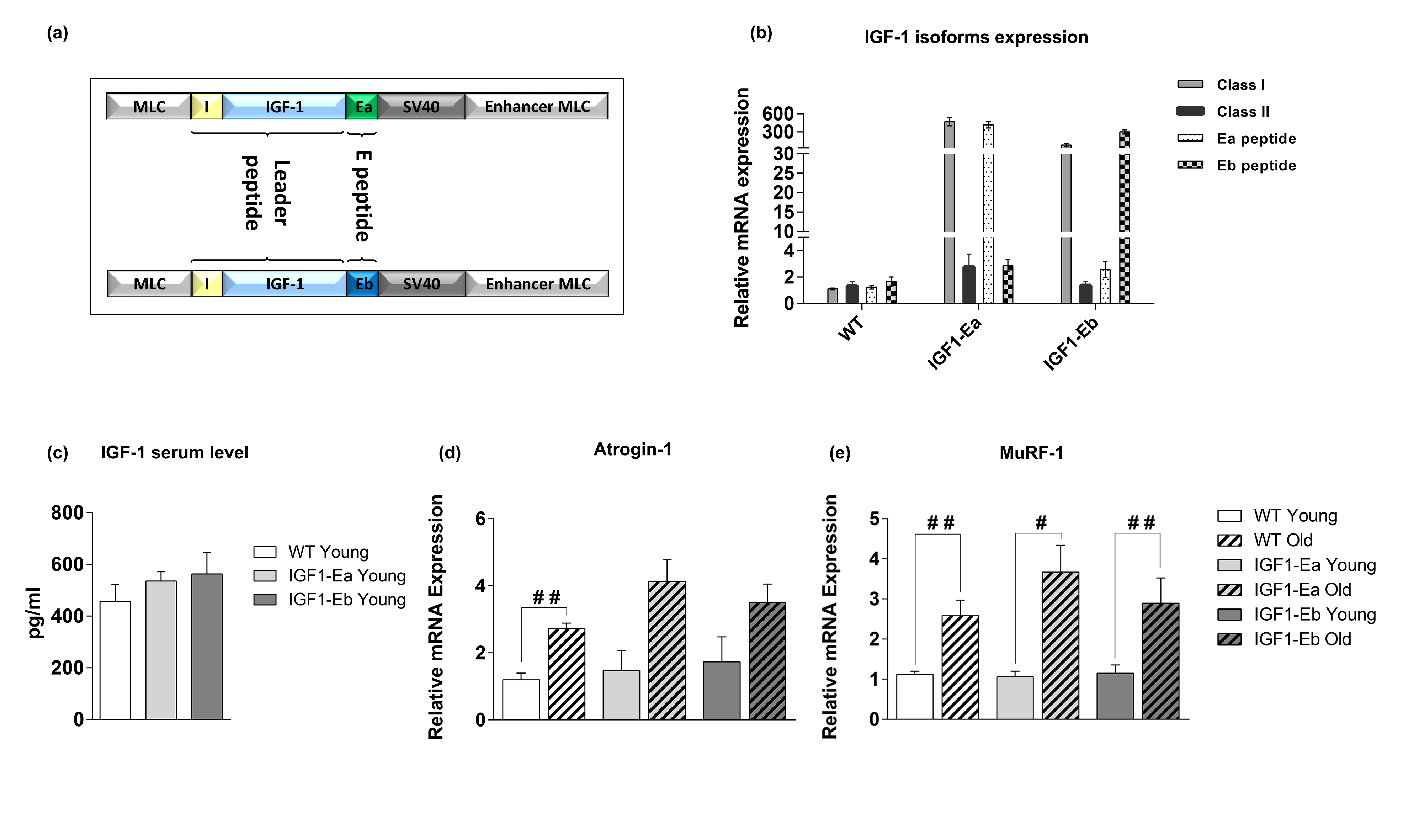

Supplement: Supplementary file 1 [file ACEL-18-e12954-s001.tif]

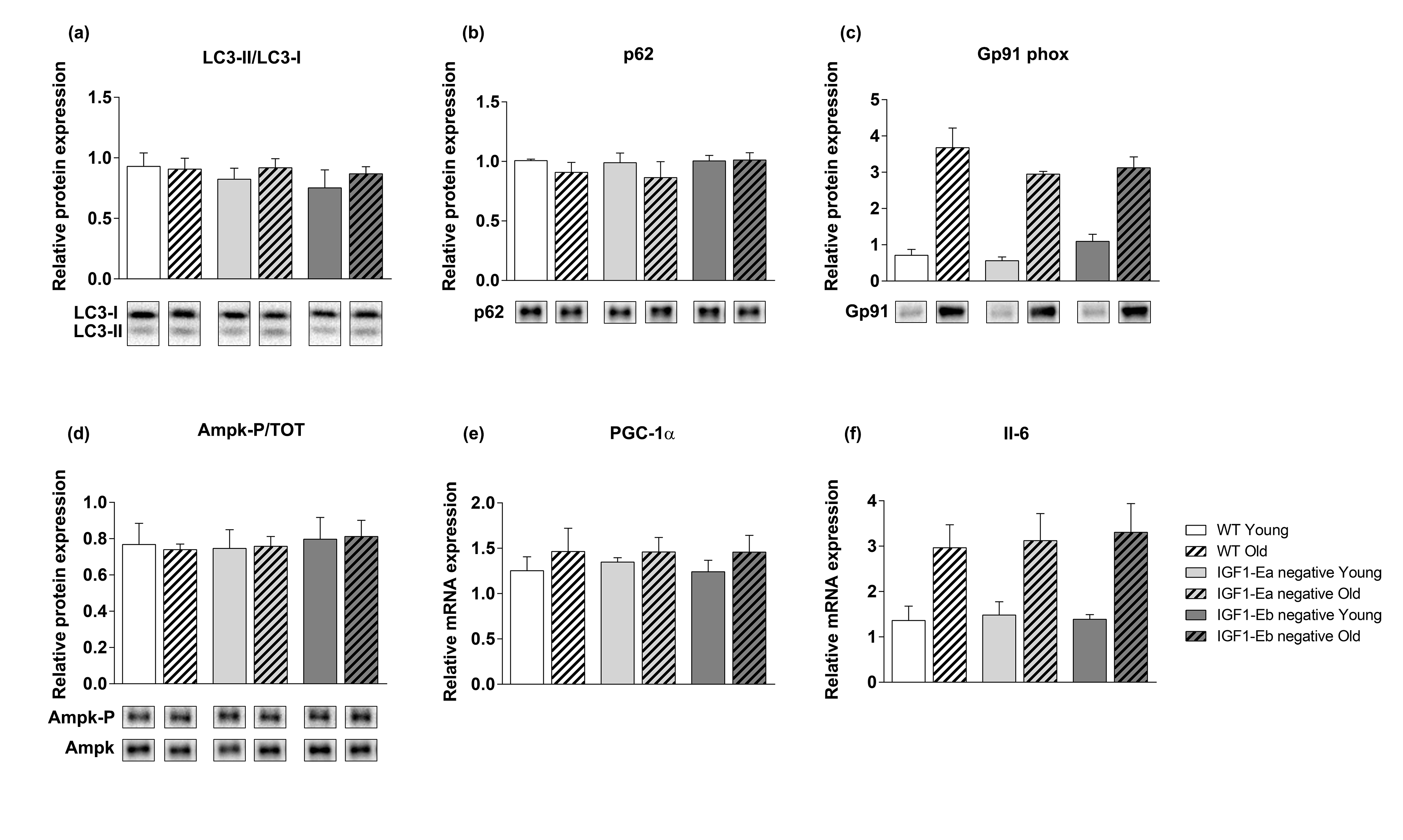

Supplement: Supplementary file 2 [file ACEL-18-e12954-s002.tif]
